# Single Session Endoscopic Removal of Bilateral Ureteric Stents Retained for Three Decades: A Case Report

**DOI:** 10.7759/cureus.4294

**Published:** 2019-03-22

**Authors:** Danny Darlington, Fatima Shirly Anitha

**Affiliations:** 1 Urology, Government Stanley Medical College and Hospital, Chennai, IND; 2 Pediatrics, Church of South India Kalyani Multispeciality Hospital, Chennai, IND

**Keywords:** double j stent, endourology, forgotten ureteric stent, single session removal, stent encrustation

## Abstract

Forgotten ureteric stents are a devastating complication, which may require a multistage approach necessitating repeated surgeries and may even require removal of the entire renal unit. We report a 59-year-old woman with heavily encrusted bilateral ureteric stents forgotten for 32 years. Successful single-stage endoscopic removal reduced the morbidity and healthcare-related costs. This case report represents the longest indwelling duration of forgotten ureteric stents reported in the literature.

## Introduction

Ureteric double J stents are an indispensable part of urological practice as they are the preferred mode of internal drainage of the urinary tract. However, they can be associated with complications such as stent encrustation, migration, infection, and stent-related lower urinary tract symptoms [1]. The most devastating complication is a forgotten stent in the urinary tract, causing loss of renal function. Forgotten double J stents usually require multiple procedures for complete removal which add to morbidity and mortality of the patient [2]. The longest indwelling time of a forgotten ureteric stent reported in the literature is 15 years [3]. We report a case of forgotten bilateral ureteric stents of 32 years duration and their successful endoscopic management using a simple and novel technique.

## Case presentation

**Figure 1:**
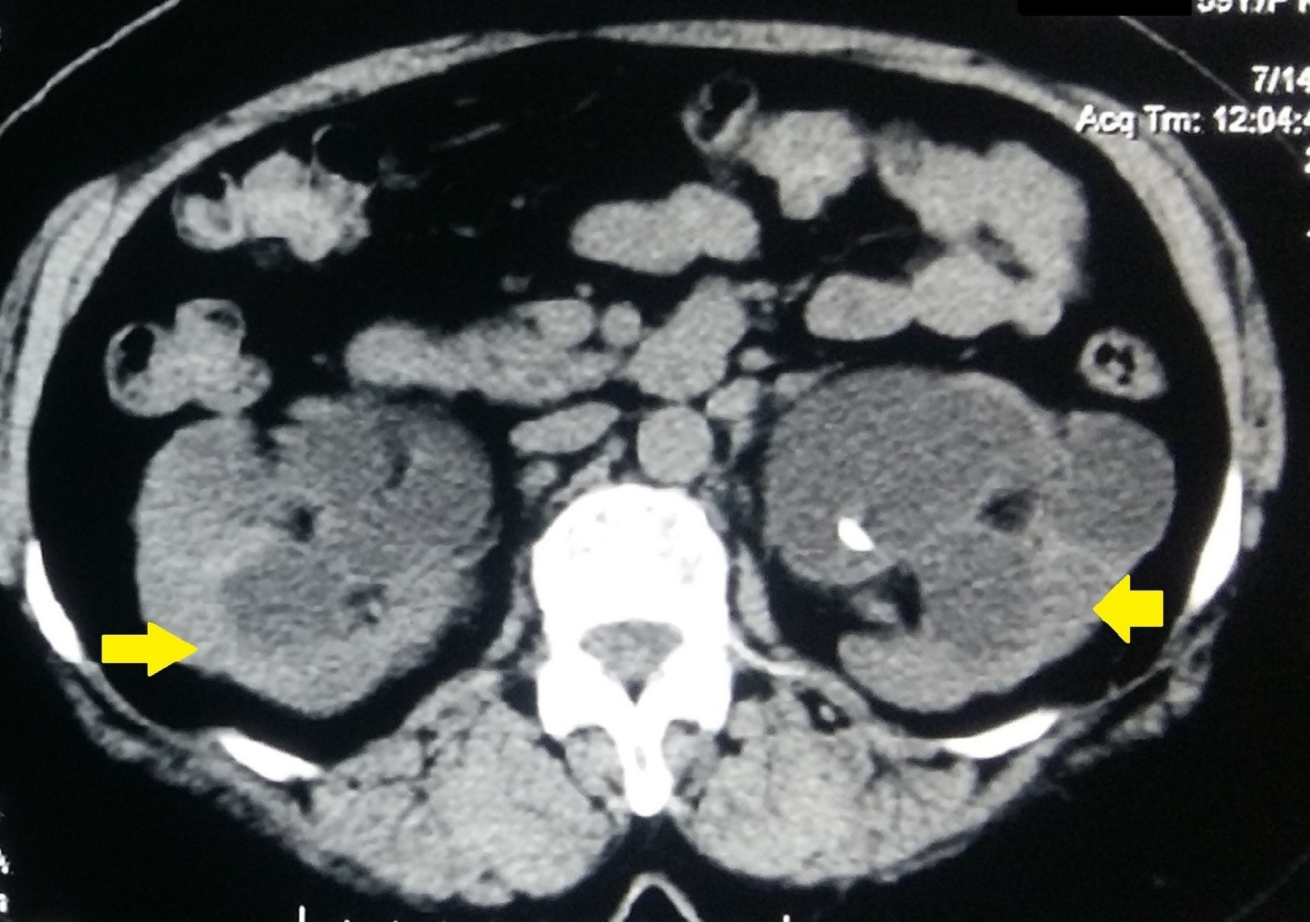
Noncontrast computed tomography showing bilateral gross hydroureteronephrosis and renal cortical thinning (yellow arrows)

**Figure 2:**
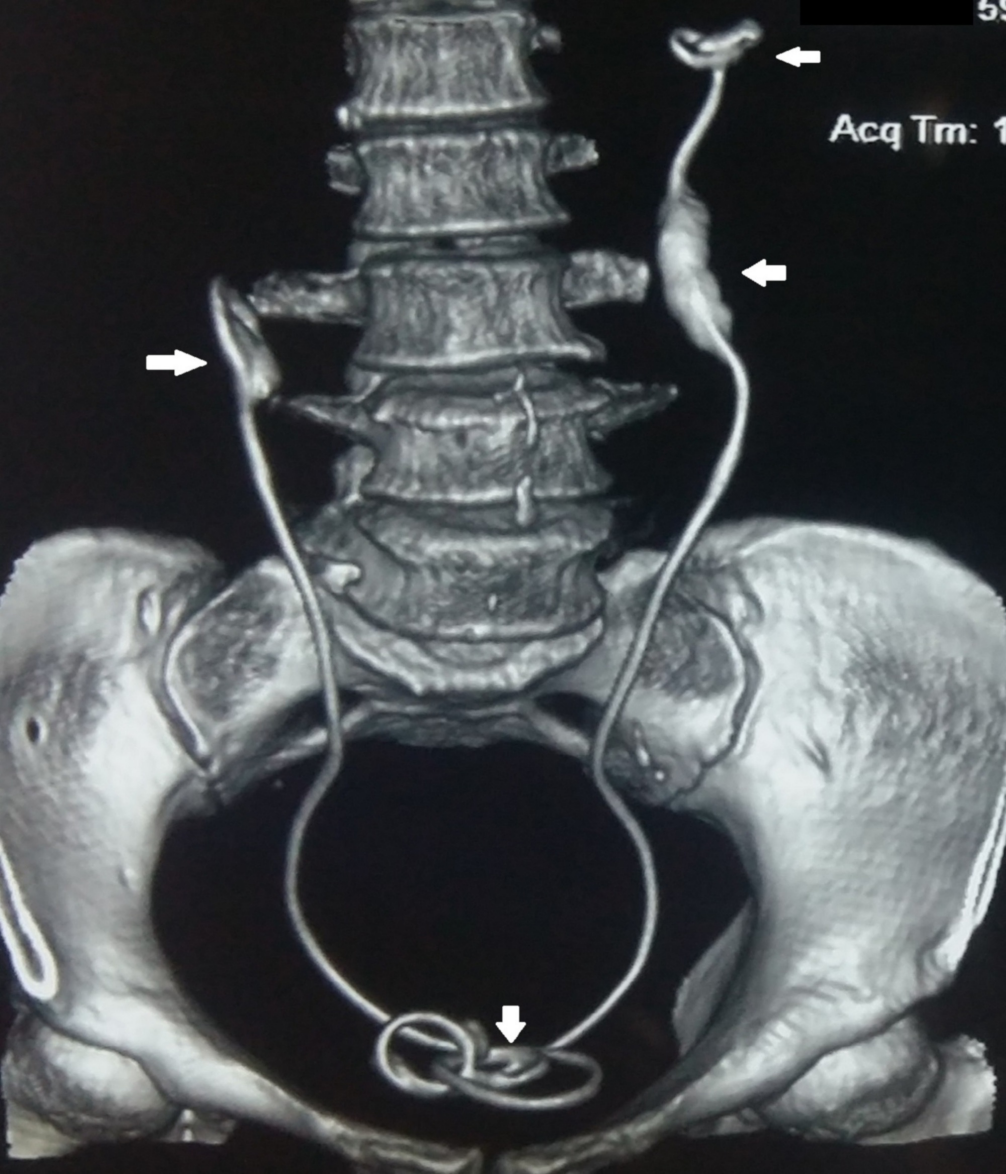
Reconstructed image of the noncontrast computed tomography depicting heavy encrustation of renal and vesical ends of both the stents (white arrows)

A 59-year-old woman presented to our outpatient department with complaints of fever and loin pain of one-week duration. She had no previous history of hematuria or loin pain and denied any history of recent instrumentation or catheterization. She had no comorbid diseases. General and systemic examinations of the patient were normal except for bilateral renal angle tenderness. Her blood urea was 80 mg/dl and serum creatinine was 1.9 mg/dl. The blood counts were within normal limits and her daily urine output was 2500 ml. Urine analysis revealed plenty of pus cells and culture of the urine revealed *Escherichia coli* and hence she was started on appropriate antibiotics. Ultrasonogram of the kidney ureter and bladder revealed bilateral gross hydroureteronephrosis with renal cortical thickness of only 5 mm. Ultrasonography also revealed bilateral double J stents in situ with associated encrustations in both the renal and vesical ends. On further probing, the patient recollected total hysterectomy performed for fibroid uterus 32 years ago for which prophylactic bilateral ureteric stent placement was performed. As she was asymptomatic, the patient never made it to remove the stents. Noncontrast computed tomography was done, which revealed bilateral gross hydroureteronephrosis with thinning of cortex in both the kidneys (Figure 1). There were bilateral ureteric double J stents with heavy encrustation in both the renal and vesical ends (Figure 2). Contrast-enhanced computed tomography was avoided on account of the persistently high renal parameters. The patient was not affordable for diuretic renogram study and hence it was decided to proceed with the removal of the stents after a course of antibiotic. We decided to remove the stents in one sitting, thereby mitigating the possibility of a forgotten stent again.

**Figure 3:**
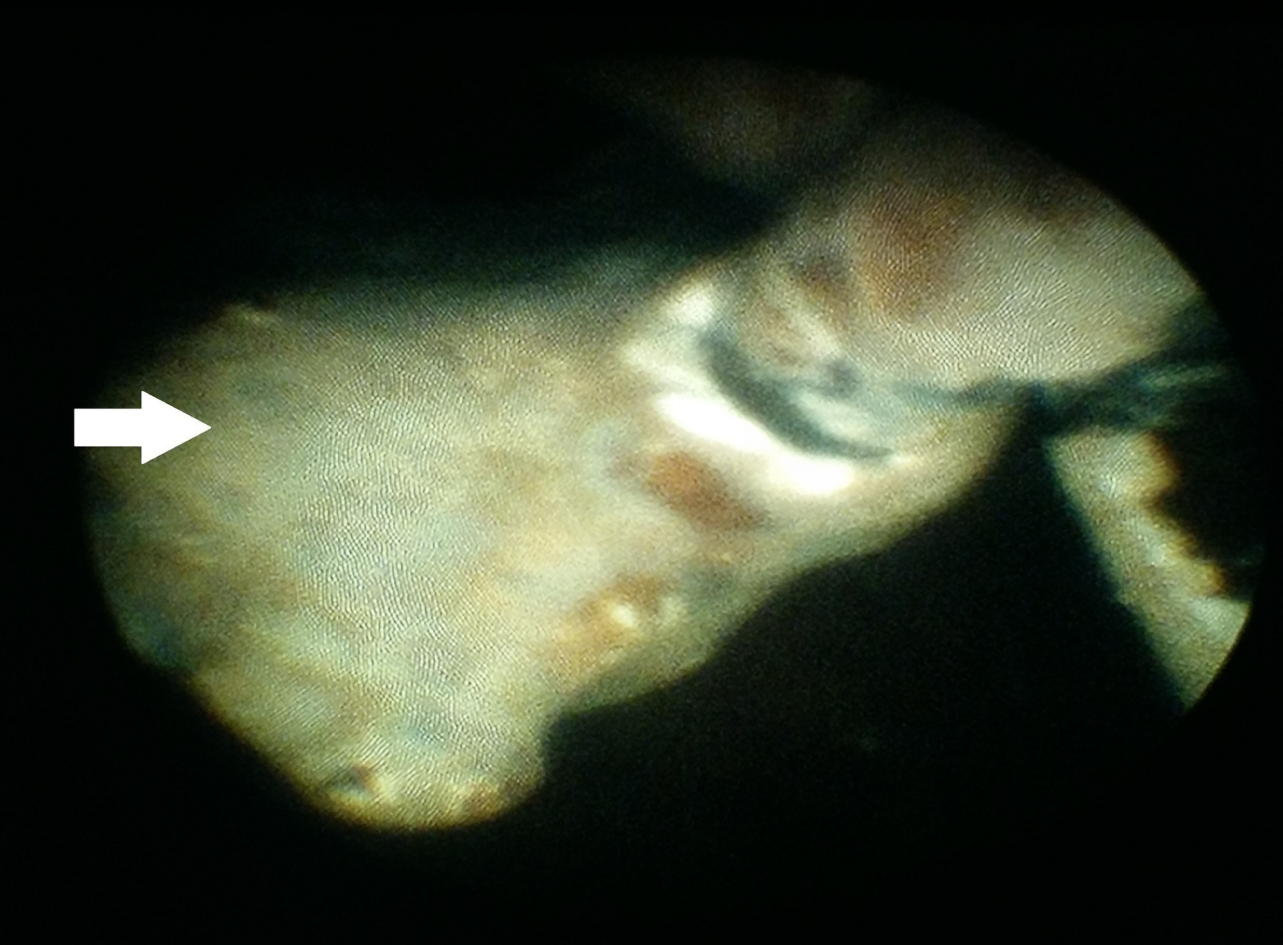
Cystoscopic view of the stone around the vesical end of the stent (white arrow)

**Figure 4:**
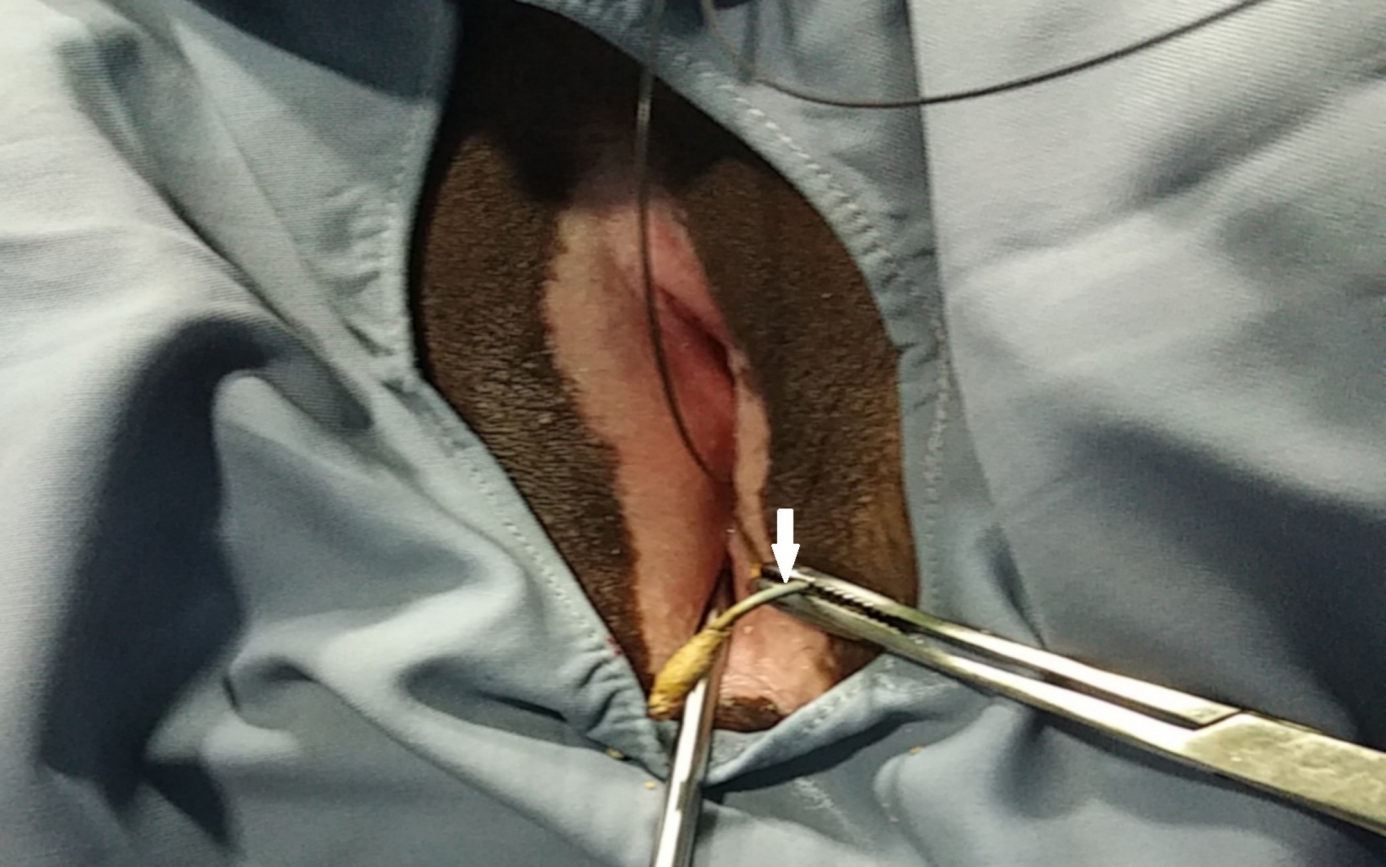
Intraoperative image showing the vesical end (white arrow) being brought out through the urethra and traction applied externally by the assistant using a hemostat

**Figure 5:**
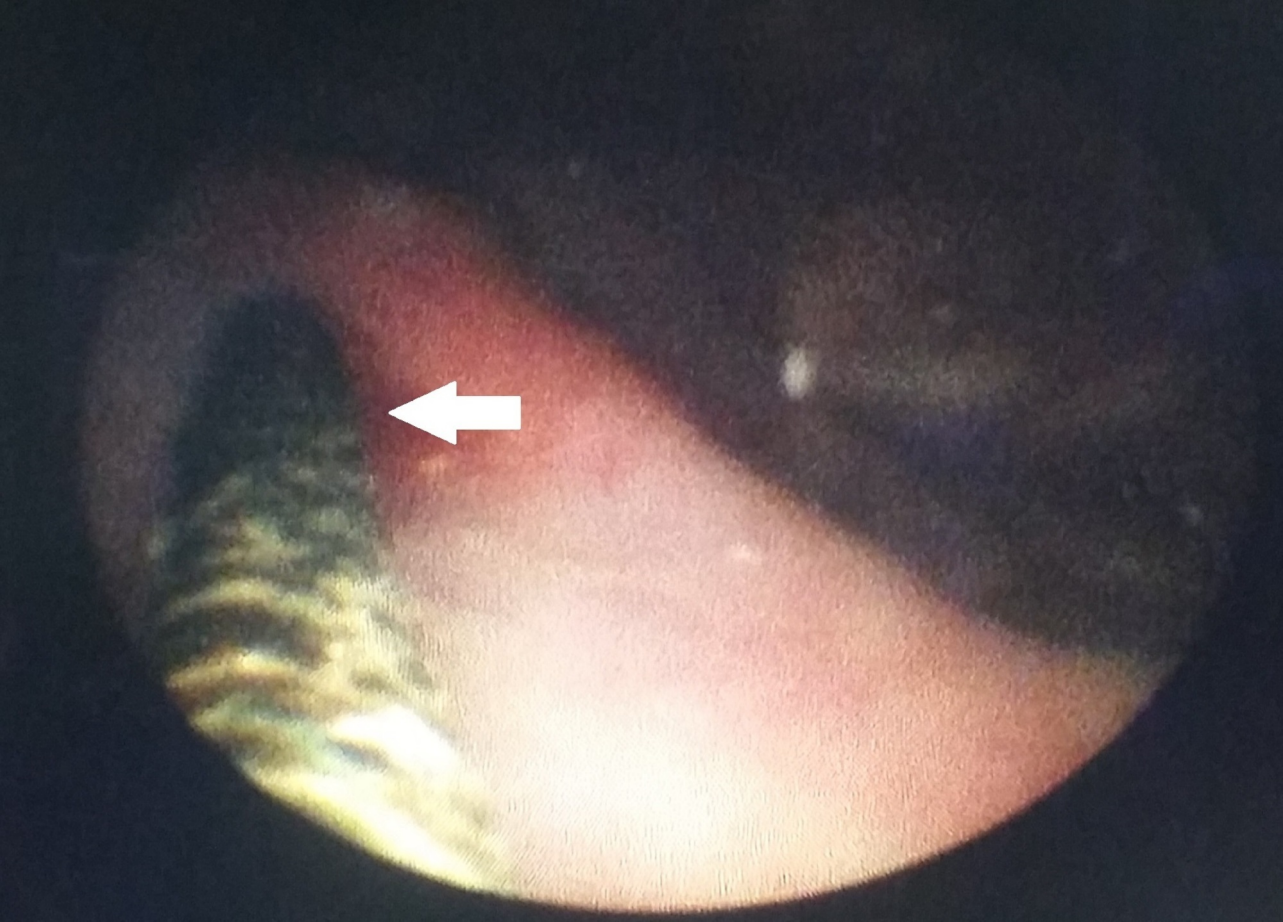
Ureteroscopic view of the right ureteric stent showing heavy encrustation of the stent at the level of the right vesicoureteric junction (white arrow)

After obtaining informed consent, the patient underwent cystoscopy that showed heavy encrustation with stones of size 3 cm around the vesical ends of the stents (Figure 3). Hence, using a stone punch, the encrustations were removed leaving the stents intact. The vesical end of one stent was uncurled and pulled out of the urethral meatus and held under traction by an assistant while a semirigid ureteroscope was inserted into the corresponding ureter (Figure 4). Ureteroscopy revealed complete encrustation of the ureteric part which was fragmented using pneumatic lithotripter working up all the way to the pelviureteric junction (Figure 5). A dilated ureter aided in passing the semirigid ureteroscope up into the renal pelvis. The renal end also had heavy encrustation with a stone of size around 3 cm, which was fragmented using pneumatic lithotripter, while the assistant maintained traction on the stent, thereby bringing the encrusted renal end into the upper ureter. This avoids stone fragments migrating back into the renal pelvis. Intravenous furosemide also was employed to aid in flushing the fragments out of the renal pelvis. The stents were extruded out once all the encrustations were fragmented and removed. The contralateral ureteric stent also had heavy encrustation in ureteric part and renal end and hence the same steps were repeated. Intraoperative fluoroscopy was done to ensure complete stone removal. The entire procedure was carried out under antibiotic cover. In view of the extensive endoscopic manipulation and edema, bilateral ureteric catheters were placed and brought out to be removed after three days during the same hospital stay. The postoperative period was uneventful with improvement in serum creatinine, which settled to a nadir value of 1.2 mg/dl.

## Discussion

Double J stents are commonly used in urological procedures for internal drainage of the urinary tract. They are deployed following endourological procedures, pyeloplasty, ureteric anastomosis and before colorectal surgeries and gynaecological surgeries like hysterectomy to aid in easy identification of the ureters. However, they are fraught with a myriad of complications such as encrustation, stent symptoms, infection, migration, and forgotten stent. The latter complication threatens the functioning of the renal unit and can even necessitate removal of the entire renal unit [4].

Ureteric stents have long been deployed prophylactically before colorectal surgeries and gynecological procedures like hysterectomy in an attempt to reduce the risk of ureteric injury. However, recent studies report that prophylactic ureteric stenting does not reduce the incidence of ureteric injury [5]. Good surgical dissection and visualization of the ureters formed the mainstay of ureteric preservation in such surgeries [6-7].

Forgotten or retained double J stents have been a nightmare for urologists as they attract both medical and legal problems to the patient and treating physician respectively [8]. Every step has been taken universally to prevent this catastrophe. Nonetheless, several cases of forgotten ureteric stents have been reported in the literature. The indwelling time of the stent has an important implication in the mode of removal of a forgotten stent [9]. The longest indwelling time of retained ureteric stent reported in the literature so far is 15 years [3]. Our case is unique in that the stent was neglected for 32 years, which could be attributed to the paucity of symptoms.

Forgotten double J stents usually are treated by the multimodal endourologic approach. The commonly performed endourological procedures in this approach are extracorporeal shockwave lithotripsy, percutaneous nephrolithotomy, retrograde semirigid ureterorenoscopy with intracorporeal lithotripsy, and cystolithalapaxy. Multiple procedures are necessary for heavily encrusted stents adding to the cost, morbidity, and mortality [2].

Recently, there have been several literature reports of single-sitting management of forgotten double J stents. Bostanci et al. reported the combined use of multiple endourological procedures in one sitting, thereby avoiding repeated anesthesia and shortening the hospital stay. They were able to achieve stone and stent free rates approaching 100% with a mean hospital stay of 3.4 ± 4.0 days [10]. The technique proposed by us works as a cost-effective method for single-step removal of encrusted stents. However, in view of the short urethra, it is easier done in women with forgotten stents than men. A dilated ureter facilitates the passage of semirigid scope up into the pelviureteric junction. Placement of ureteric catheter aids in easy removal during the same hospital stay and avoids another episode of a forgotten stent episode in noncompliant patients.

As prevention is better than cure steps must be taken by the surgical team to ensure timely removal of all ureteric stents. Ziemba et al. have reported a point of care mobile application called ureteral stent tracker which can be used to log stent insertion, schedule the removal of stents and confirm the same [11]. Another method is the use of a computerized system that tracks stents and automatically emails the physician about the schedule for removal of stent [12].

## Conclusions

This case report has been presented for the longest indwelling period and the completely asymptomatic nature of the forgotten stent which delayed its removal. A novel technique of single-session removal of encrusted double J stents has been described. Elaborate studies are warranted in the future to analyse the cost effectiveness and clinical utility of this simple technique of stent removal.
